# Comparison of Anterior Segment Biometric Measurements between Pentacam HR and IOLMaster in Normal and High Myopic Eyes

**DOI:** 10.1371/journal.pone.0143110

**Published:** 2015-11-17

**Authors:** Jing Dong, Maolong Tang, Yaqin Zhang, Yading Jia, Haining Zhang, Zhijie Jia, Xiaogang Wang

**Affiliations:** 1 The First Hospital of *Shanxi Medical University*, Shanxi, P.R. China; 2 Casey Eye Institute, Oregon Health and Science University, Portland, Oregon, United States of America; 3 Shanxi Eye Hospital, Shanxi, P.R. China; Bascom Palmer Eye Institute, University of Miami School of Medicine;, UNITED STATES

## Abstract

**Purpose:**

To compare the anterior chamber depth (ACD), keratometry (K) and astigmatism measurements taken by IOLMaster and Pentacam HR in normal and high myopic (HM) eyes.

**Design:**

A prospective observational case series.

**Methods:**

Sixty-six normal eyes and 59 HM eyes underwent ACD, keratometry and astigmatism measurements with both devices. Axial length (AL) was measured on IOLMaster. The interdevice agreement was evaluated using the Bland-Altman analysis and paired t-test. The correlations between age and AL & ACD were analyzed. Vector analysis was used to compare astigmatism measurements.

**Results:**

The ACD from IOLMaster and Pentacam HR was different for the normal group (P = 0.003) but not for the HM group (P = 0.280). IOLMaster demonstrated higher steep K and mean K values than Pentacam HR for both normal and HM groups (P<0.001 for all). IOLMaster also have higher flat K values for the HM groups (P<0.001) but were statistically equivalent with Pentacam HR for the normal group (P = 0.119) IOLMaster and Pentacam HR were different in astigmatism measurements for the normal group but were statistically equivalent for the HM group. For the normal group, age was negatively correlated with AL, IOLMaster ACD and Pentacam HR ACD (r = -0.395, P = 0.001; r = -0.715, P < 0.001; r = -0.643, P < 0.001). For the HM group, age was positively correlated with AL but negatively correlated with IOLMaster ACD and Pentacam HR ACD (r = 0.377, P = 0.003; r = -0.392, P = 0.002; r = -0.616, P < 0.001).

**Conclusions:**

The IOLMaster and Pentacam HR have significant difference in corneal power measurements for both normal and HM groups. The two instruments also differ in ACD and astigmatism measurement for the normal group. Therefore, a single instrument is recommended for studying longitudinal changes in anterior segment biometric measurements. Age should be considered as an influencing factor for both AL and ACD values in the normal and HM group.

## Introduction

For clinical applications, accurate anterior chamber depth (ACD) and anterior corneal power measurements are important for the design and ultimate success of vision correction, including refractive and cataract surgery, especially for high myopia (HM).[1–3] Currently, a number of instruments are available to measure anterior segment biometry, including Scheimpflug topography, optical coherence tomography, optical low-coherence reflectometry, partial coherence interferometry and slit-scanning topography/pachymetry systems.[4–7]

The Pentacam (OCULUS, Wetzlar, Germany), which uses a single rotating Scheimpflug camera (180°) and monochromatic slit-light source (blue LED at 470 nm) combined with a static camera, can provide a 3-dimensional model of the anterior segment. The ACD and anterior corneal keratometry measurements generated by the Pentacam have been shown to have excellent repeatability.[8] There is a special 3D high-resolution (HR) scanning mode, in which the camera takes 50 images in 1 second and 138,000 true elevation points are evaluated. This mode was claimed to provide better image quality with optimized optics and new software features like contact lens fitting and 3D pIOL simulation. In this study, the HR mode was used (referred to as Pentacam HR).

The IOLMaster (Carl Zeiss Meditec, Germany) is partial coherence interferometer used for anterior segment measurements. It measures the anterior corneal keratometry using the data from six light reflections oriented in a hexagonal pattern approximately 2.3 mm diameter. It can provide highly repeatable values such as the corneal power, corneal astigmatism, ACD and axial length (AL), and these parameters are vital for intraocular lens (IOL) power calculation and planning IOLs implantation.[9,10]

Current literature has not established whether the ACD, astigmatism and keratometry values of these two devices are interchangeable in HM. The purpose of this study was to compare the ACD, corneal keratometry and astigmatism measurement with IOLMaster and Pentacam HR in normal and HM patients.

## Materials and Methods

This study was performed at the Shanxi Eye Hospital (Taiyuan, Shanxi, China). The research protocols were approved by the institutional review boards in Shanxi Eye Hospital and carried out in accordance with the tenets of the Declaration of Helsinki. Written informed consent was obtained from each subject after they were given an explanation of the nature of the study. For age less than 18 years or more than 70 years, the written informed consent was obtained from their legal guardian.

### Subjects

We just chose Han Chinese subjects to eliminate the possible influences of different ethnic groups. The normal and HM subjects were chosen from the Ophthalmic Clinic Center at the Shanxi Eye Hospital. One random eye of each subject was chosen for both devices. The inclusion criteria for the normal group included: a best-corrected visual acuity (BCVA) of ≥ 16/20, a refractive error < 3 diopter (D) spheres, normal slit-lamp and fundoscopy examinations, an intraocular pressure (IOP) < 22 mmHg, and no history of ocular or systemic corticosteroid use. The inclusion criteria for the HM subjects included: a BCVA of ≥ 20/80, a spherical refractive error more negative than -6 diopters and axial length (AL) > 26 mm, and central fixation sufficiently stable to perform image capture. Subjects with keratoconus, previous corneal lesions and prior surgery in the cornea, severe cataracts, glaucoma or posterior abnormalities, such as choroidal neovascularization, retinoschisis, retinal detachment or macular holes, were excluded.

### Data Acquisition

The ACD, keratometry, and corneal astigmatism were measured on Pentacam HR then IOLMaster. Each measurement was repeated three times in each eye and the averaged value was used in the analysis (individual participants’ data are presented in S1 Dataset). The software was version 1.20r36 for Pentacam HR and 7.5 for IOLMaster. The subject was asked to place his chin on the chin rest, and press his forehead against the forehead strap. The subject’s eye was aligned to the visual axis by a central fixation light or target. A single trained operator performed all of the examinations using both devices. The keratometry index was 1.3375 and the ACD value was the distance from the corneal epithelium to the anterior lens surface.

### Vector Analysis of Astigmatism

Vector analysis and double-angle plots were used to compare the corneal astigmatism from the two devices.[11] The astigmatism values was decomposed into two perpendicular components as follows:

X=A cos(2α); (1A)

Y=A sin(2α) (1B)

Where

X = cardinal component,

Y = oblique component,

A = astigmatism magnitude in diopters,

α = astigmatism axis in degrees.

### Statistical Analysis

Statistical analysis was performed with SPSS ver. 13.0. To compare age, ACD and AL variables in the normal and HM eyes, independent sample t-tests were used. The Chi-Square test was performed to compare the percentile differences between groups. The correlation coefficients were also calculated for age, ACD and AL in both groups. Vector analysis was used to compare corneal astigmatism measurements between the two devices.^11^ The statistical significance of the interdevice differences in ACD, anterior corneal keratometry and astigmatism parameters was evaluated with the paired two-tailed t-test. Inter-device agreement was evaluated using Bland-Altman analysis. The inter-device differences were plotted against their means, and the 95% limits of agreement (LoA) were determined using this method. The significance level for all of the tests was set at 5%.

## Results

A total of 59 eyes from 59 HM subjects and 66 eyes from 66 normal subjects were included in the study (Table 1). The subjects’ ages ranged from 5 to 89 years and 5 to 79 years for the normal and HM groups, respectively. The two groups were well-matched for age and gender.

**Table 1:** Characteristics of the study groups.

	Normal Group	High myopia Group	*P* Value*
Patients, n	66	59	
Eyes, n	66	59	
Age (yrs)	43 ± 29	36 ± 25	0.163
Gender (male/female)	23/43	27/32	**0.214**
AL (mm) ^#^	23.09± 0.87	28.16±2.49	<0.001
ACD (mm, by IOLMaster) ^#^	3.28± 0.45	3.66 ± 0.41	<0.001
ACD (mm, by Pentacam HR) ^#^	3.37± 0.52	3.69± 0.42	<0.001
Flat K (D, by IOLMaster) ^#^	44.21±1.42	42.60±1.41	<0.001
Flat K (D, by Pentacam HR) ^#^	44.16±1.46	42.51±1.44	<0.001
Steep K (D, by IOLMaster) ^#^	45.37±1.47	43.92±1.47	<0.001
Steep K (D, by Pentacam HR) ^#^	45.19±1.48	43.81±1.44	<0.001
Mean K (D, by IOLMaster) ^#^	44.80±1.40	43.26±1.39	<0.001
Mean K (D, by Pentacam HR) ^#^	44.67±1.42	43.16±1.40	<0.001
Astigmatism Magnitude (D, by IOLMaster)	1.16±0.75	1.32±0.74	0.233
Astigmatism Magnitude (D, by Pentacam HR) ^#^	1.02±0.73	1.29±0.70	0.035

ACD = anterior chamber depth, AL = axial length.

*All calculated by independent sample t test, except the values in bold, which was calculated by chi-square test.

^#^ Statistical significant using the significance level at 5%.

**Fig 1:**
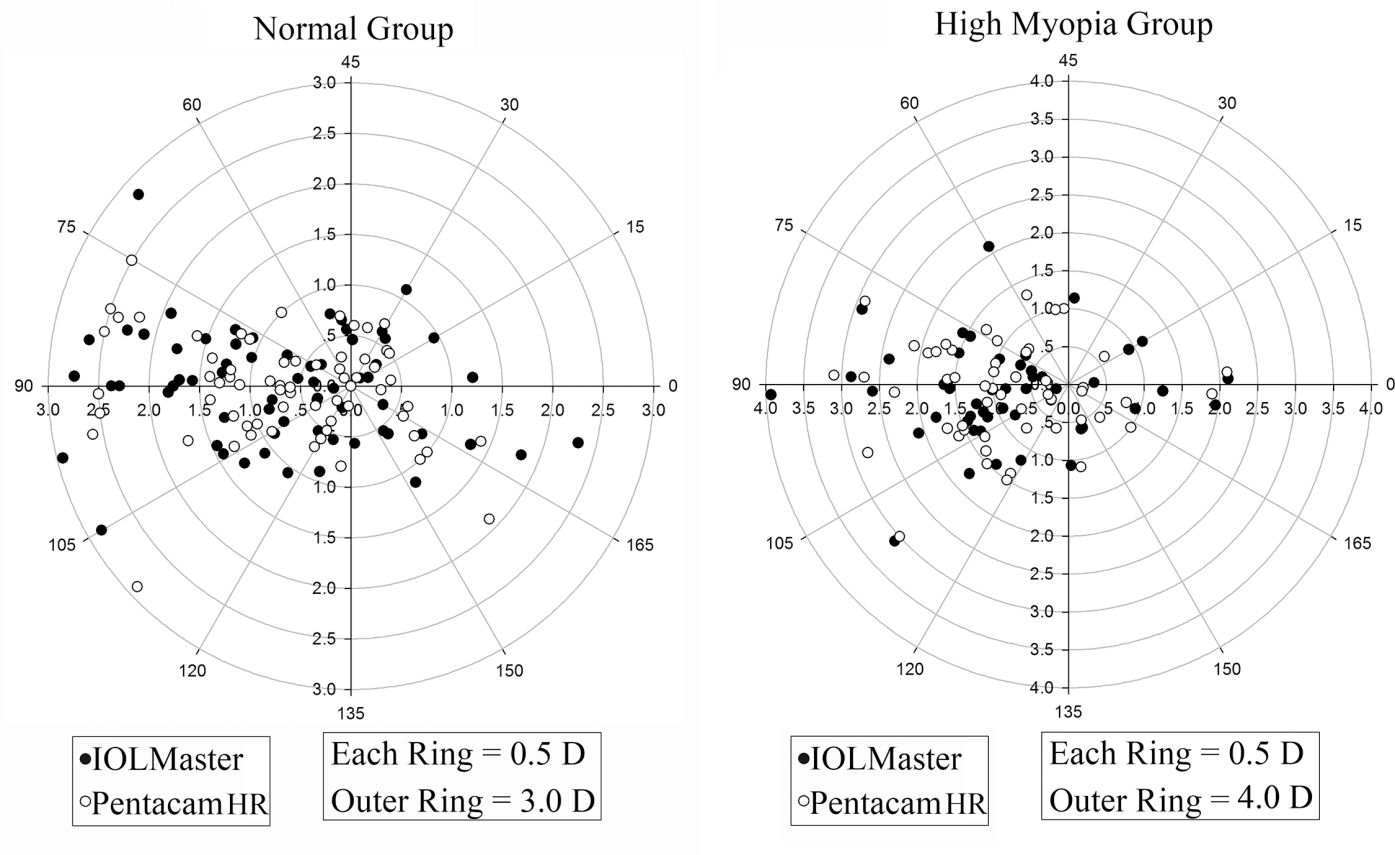
Double-angle plot of corneal astigmatism in the normal and high myopia group. Most eyes have with-the-rule astigmatism (axis at 90°). The corneal astigmatism measurement was equivalent between IOLMaster and Pentacam HR for the HM group. However, the astigmatism magnitude was lower in Pentacam HR compared to IOLMaster by 0.14D in the normal group (p = 0.002).

The mean AL and ACD readings of HM were significantly higher than those of the normal group (P < 0.001 for all; Table 1). The mean differences for corneal power were all less than 0.2D in both groups. Using magnitude analysis, Pentacam HR exhibited significantly lower steep K, mean K than IOLMaster in both normal and HM group (Table 2). The corneal astigmatism measurement was equivalent between IOLMaster and Pentacam HR for the HM group. However, the astigmatism magnitude was statistically lower in Pentacam HR compared to IOLMaster by 0.14D in the normal group (Table 2). Fig 1 showed the distribution of corneal astigmatism of both groups in double-angle plots. Moreover, neither the corneal astigmatism magnitude nor the cardinal/oblique components showed statistical significance between men and women in either group.

**Table 2:** Anterior chamber depth, anterior corneal keratometry and astigmatism data comparison between IOLMaster and Pentacam HR in the normal group and high myopia group.

	Normal Group				High Myopia Group			
	IOLMaster	Pentacam HR	Difference (I—P)	*P* *	IOLMaster	Pentacam HR	Difference (I—P)	*P* *
ACD (mm) ^#^	3.28± 0.45	3.37± 0.52	-0.08±0.22	0.003	3.66 ± 0.41	3.69± 0.42	-0.03±0.21	0.280
Flat K (D) ^#^	44.21±1.42	44.16±1.46	0.06±0.30	0.119	42.60±1.41	42.51±1.44	0.09±0.18	0.001
Steep K (D) ^#^	45.37±1.47	45.19±1.48	0.18±0.30	<0.001	43.92±1.47	43.81±1.44	0.11±0.24	0.001
Mean K (D) ^#^	44.80±1.40	44.67±1.42	0.12±0.24	<0.001	43.26±1.39	43.16±1.40	0.10±0.16	<0.001
Astigmatism Magnitude (D) ^#^	1.16±0.75	1.02±0.73	0.14±0.35	0.002	1.32±0.74	1.29±0.70	0.02±0.27	0.483
Astigmatism Cardinal	-0.65± 1.09	-0.62± 0.95	-0.03±0.43	0.607	-0.81±1.13	-0.83±1.03	0.02±0.29	0.568
Astigmatism Oblique	-0.02± 0.55	-0.03± 0.52	0.02±0.32	0.700	-0.08±0.60	-0.13±0.62	0.05±0.34	0.262

D = diopter; I = IOLMaster; K = keratometry; P = Pentacam HR.

*Paired two-tailed t-test.

^#^ Statistical significant using the significance level at 5% for either normal or HM group.

**Fig 2:**
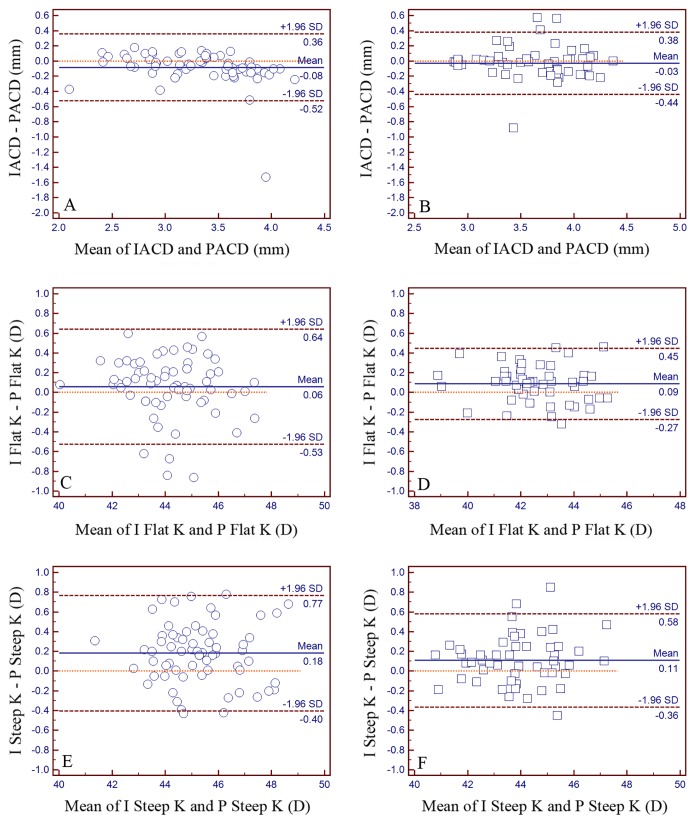
Differences in mean anterior chamber depth (ACD), flat keratometry (Flat K) and steep keratometry (Steep K) between IOLMaster (I) and Pentacam HR (P). Pentacam HR showed higher ACD values than IOLMaster in the normal group (P = 0.003). The interdevice difference in ACD measurement was not statistically significant in the high myopia group (P = 0.280). IOLMaster demonstrated higher steep K than Pentacam HR for both normal and high myopia groups (P<0.001). IOLMaster also have higher flat K values for the HM groups (P<0.001) but agreed with Pentacam HR for the normal group (P = 0.119). Panel A, C, E for the normal group and Panel B, D, F for the high myopia group.

Pentacam HR showed higher ACD values than IOLMaster in the normal group (P = 0.003). The interdevice difference in ACD measurement was not statistically significant in the HM group (P = 0.280). The inter-device 95% LoA range for the ACD, flat K and steep K values in the normal and HM groups were 0.88 mm and 0.82 mm, 1.17D and 0.72D, 1.17D and 0.94D, respectively (Fig 2).

**Fig 3:**
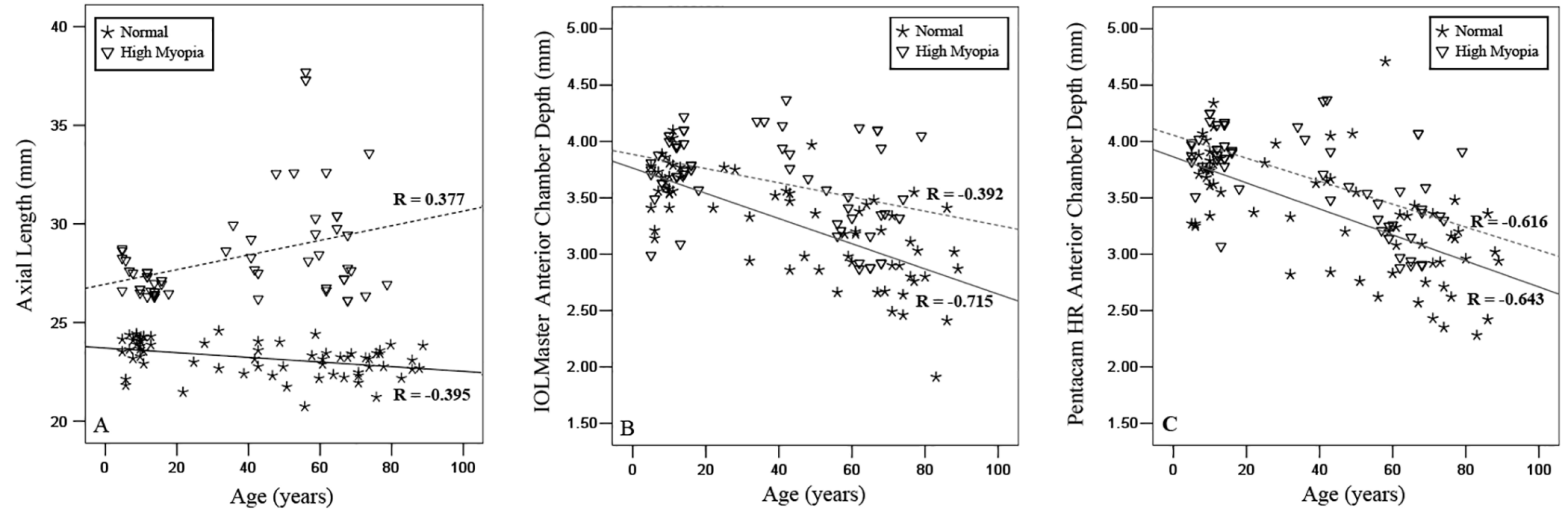
Plots of axial length versus age and anterior chamber depth (ACD) versus age in both normal and high myopia groups. The linear regression trend lines are solid in the normal group and dotted in the high myopia group. The ACD values of IOLMaster and Pentacam HR were demonstrated in panel B and panel C, respectively. For the normal group, age was negatively correlated with AL, IOLMaster ACD and Pentacam HR ACD (r = -0.395, P = 0.001; r = -0.715, P < 0.001; r = -0.643, P < 0.001). In the HM group, age was positively correlated with AL but negatively correlated with IOLMaster ACD and Pentacam HR ACD (r = 0.377, P = 0.003; r = -0.392, P = 0.002; r = -0.616, P < 0.001).

A Pearson correlation analysis showed that for the normal group, age was negatively correlated with AL, IOLMaster ACD and Pentacam HR ACD (r = -0.395, P = 0.001; r = -0.715, P < 0.001; r = -0.643, P < 0.001; Fig 3). For the HM group, age was positively correlated with AL but negatively correlated with IOLMaster ACD and Pentacam HR ACD (r = 0.377, P = 0.003; r = -0.392, P = 0.002; r = -0.616, P < 0.001; Fig 3).

For normal group, both IOLMaster ACD and Pentacam HR ACD showed positive correlation with AL (r = 0.497, P<0.001; r = 0.508, P<0.001). For HM group, Pentacam HR ACD showed negative correlation with AL (r = -0.289, P = 0.026). The IOLMaster ACD also had negative correlation with AL but it was not statistically significant (r = -0.206, P = 0.117).

## Discussion

The requirement of precise and accuracy measurement of anterior segment characteristics mandates the development of reliable measurement devices, especially non-contact instruments. It is essential to compare the inter-devices interchangeability in practice. This research evaluated the comparability of anterior biometric measurements between IOLMaster and Pentacam HR in normal and HM subjects. We found that the IOLMaster and Pentacam HR had statistically significant difference in corneal power measurements for both normal and HM groups. The two devices agreed on astigmatism measurement for HM group. However, the astigmatism magnitudes from the two devices differ in the normal group. ACD measurements obtained by IOLMaster differed significantly from those obtained by Pentacam HR in the normal group but not in the HM group.

The poor agreement for ACD values in the normal group agrees with some previous studies.[12–16] The ACD measurement discrepancy may attribute to the different optic methods for ACD measurement and the potential off-axis measurement. For IOLMaster, the ACD measurement could be influenced by systematic errors due to the distortion effects in optic media with different refractive indices. For Pentacam HR, the ray tracing technology can compensate that problem.[15] The observed mean error of 0.09 mm between the two devices was too small to create any noticeable difference in refractive outcome.[17] On the other hand, no statistical difference of ACD measurement was found for HM group. We speculated that the difference in ACD for normal eyes might be from the errors due to accommodative changes induced by the instruments. Because HM eyes tend to have less accommodative changes compared to normal eyes [18] thus less error, the difference in ACD values between the two instruments diminished.

For the normal group, The results showed that significant difference for steep K values but not for flat K measurement between IOLMaster and Pentacam HR. This finding agreed with some previous studies.[16,19] For the HM group, both flat K and steep K were significantly higher than those from Pentacam HR. We speculated that the difference of corneal power measurement between the two instruments might be due to several factors: 1) difference analytical zone: the analyzing area of the Pentacam HR K (Sim-K) is about 3.0 mm in diameter while IOLMaster measures corneal power over approximately 2.3mm diameter area. 2) the device optimization for Pentacam: Tang et al. has demonstrated that the Pentacam version 1.16r04 Scheimpflug corneal power measurements were consistently steeper than the true corneal power. [20] Moreover, Karunaratne N et al. has showed that constant optimization may be a necessary way to minimize the systemic differences between keratometric devices. [21] For the comparison of corneal astigmatism in normal eyes, Delrivo et al found the statistical difference in the J_0_ but not in the J_45_ vector using the Jackson cross cylinder notation J_0_ and J_45._[22,23] However, in the present study, neither astigmatism cardinal nor oblique component demonstrated significant difference between the two devices in both normal and HM group but the astigmatism magnitude of IOLMaster was 0.14D higher than Pentacam HR in normal group. Similar findings were found in the Mao et al.’s research between the Keratograph and Pentacam or IOLMaster.[24]

Similar to some previous study using Scheimpflug imaging system, ACD was negatively correlated with age in this study.[25,26] The increasing lens thickness with advancing age may account for this decrease in ACD.[27] This finding is also similar to Tuft et al.’s finding of increased AL with decreasing age at the time of cataract surgery.[28] Moreover, our previous study with Lenstar also confirmed this trend.[29] The positive correlation between age and AL in HM patients demonstrated that a progression of posterior staphyloma with increasing age is a key factor in the continuous increase of AL in adults with HM.[30]One limitation of this study is the staphyloma in the HM group, which may have an impact on AL measurements because the most posterior portion of the globe may not correspond with the center of macula. The effect of staphyloma might be minimized by the use of the fixation target.

In conclusion, the IOLMaster and Pentacam HR have statistically significant difference in corneal power measurements for both normal and HM groups. The two instruments agree on astigmatism measurement only for the HM group. Therefore, a single instrument is recommended for studying longitudinal changes in corneal power and corneal astigmatism.

## Supporting Information

S1 Dataset Baseline demographic data of this study. (XLS)
